# Exploring Patient Perspectives on the Use of Artificial Intelligence to Inform Joint Decision-Making for Patients With Multiple Conditions in Primary Care in the United Kingdom: Qualitative Study

**DOI:** 10.2196/87507

**Published:** 2026-04-21

**Authors:** Sarah Flanagan, Charlotte Spurway, Louise Jackson, Jenny Cooper, Francesca L Crowe, Shamil Haroon, Tom Marshall, Leah Fitzsimmons, Eleanor Hathaway, Krishnarajah Nirantharakumar, Thomas Jackson, Sheila Greenfield

**Affiliations:** 1 Department of Applied Health Science, University of Birmingham, Birmingham, England, United Kingdom; 2 Health Economics Unit, University of Birmingham, IOEM Building, Birmingham, England, United Kingdom, 44 01214146486; 3 Department of Metabolism and Systems Science, University of Birmingham, Birmingham, England, United Kingdom; 4 Department of Inflammation and Ageing, University of Birmingham, Birmingham, England, United Kingdom

**Keywords:** artificial intelligence, AI, multiple long-term conditions, qualitative research, primary care

## Abstract

**Background:**

Multimorbidity, living with 2 or more long-term health conditions, is increasing globally and now affects over one-quarter of adults in England. People with multiple long-term conditions (MLTC) face complex health and treatment challenges, often experiencing fragmented care within systems oriented toward single-disease management. Artificial intelligence (AI) has the potential to support clinicians and patients by analyzing complex health data, optimizing treatment strategies, and predicting disease trajectories.

**Objective:**

The OPTIMAL (Optimizing Therapies, Disease Trajectories, and AI-Assisted Clinical Management for Patients Living with Complex Multimorbidity) project aims to develop AI-enabled tools to support shared decision-making in primary care. This study explored how patients with MLTC perceive the use of AI to inform joint decision-making in primary care.

**Methods:**

Semistructured interviews were conducted via telephone or video call with 29 adults living with MLTC between July and November 2023. Participants were recruited through general practitioner practices via the Clinical Practice Research Datalink and community-based organizations across the West Midlands. Interviews were transcribed verbatim and analyzed thematically using an inductive approach. Members of a patient advisory group were involved in developing study materials, refining the interview guide, and reviewing emerging findings to ensure relevance and authenticity.

**Results:**

Participants identified potential benefits of AI in enhancing consultation efficiency and accuracy, improving access to information for patients and clinicians, promoting early detection of health changes, and reducing health care inequalities. However, concerns were raised about the loss of human interaction, data privacy and security, transparency of algorithms, and the potential for bias and inequity in AI systems. Trust and acceptance varied by age and familiarity with technology. Some participants expressed uncertainty about what AI entails and how it could be used in primary care.

**Conclusions:**

Patients with MLTC viewed AI-assisted decision-making in primary care with cautious optimism. While many recognized potential benefits for coordination and personalization of care, others expressed reservations about privacy, fairness, and the risk of diminished human connection.

## Introduction

### Background

The prevalence of multimorbidity (living with 2 or more long-term health conditions) is increasing worldwide and affects over a quarter of adults in England [1,2]. Patients with multiple long-term conditions (MLTC) experience unique challenges that can impact their quality of life and functional status. More widely, complex MLTC, defined as the coexistence of 4 or more long-term health conditions, has a significant contribution to health care utilization and often leads to polypharmacy [3].

As mainstream services are generally oriented to a “single disease model” of care, costs and service utilization generally increase as the number of conditions a patient has increases [4,5]. Conventional approaches to treating individual conditions in isolation fall short when managing complex MLTC due to the unique complexities, which have implications for experiences of care, costs borne by patients, and impacts on work and other activities [6]. Traditional clinical trials, which typically inform evidence-based guidelines, often exclude individuals with MLTC, resulting in guidelines that do not adequately address the intricacies of complex MLTC [7]. This limitation underscores the critical need for innovative approaches to guide treatment decisions.

Artificial intelligence (AI) has been seen to offer potential solutions to this dilemma, offering the capability to navigate the intricate web of multiple conditions and varied treatments. Definitions of AI have progressed over time and generally relate to machines and algorithms that can “reason and adapt based on sets of rules and environment which mimic human intelligence” [8]. AI tools possess the capacity to interpret vast and varied datasets, accounting for individual patient characteristics and medical histories in ways that can exceed human capabilities [9]. In particular, AI tools can be used as part of the consultation process to inform clinician and patient decision-making around diagnoses, planning treatments, and thinking about outcomes, although the use in primary care for the management of patients to date has been limited [10,11]. This potential is particularly relevant in the context of complex MLTC, where tailoring treatment plans to individual patients becomes increasingly challenging. The OPTIMAL (Optimizing Therapies, Disease Trajectories, and AI-Assisted Clinical Management for Patients Living with Complex Multimorbidity) project aims to leverage AI to develop a joint decision-making tool specifically designed for primary care settings, aimed at identifying the most effective treatment strategies with the lowest risk of additional side effects or antagonizing current conditions and predicting disease trajectories in individuals with MLTC [12].

### Objectives

Existing research on patient perceptions in relation to AI in health care has highlighted that while AI tools are generally welcomed, there are a range of concerns. These include potential impacts on the quality of health care received, implications for privacy and data protection, and the accuracy of any AI outputs [13,14]. Hence, it is important to involve patients in developing and implementing AI in health care to ensure that solutions are acceptable and enhance patient care and experiences. Through semistructured interviews, this study sought to capture insights into how AI-informed joint decision-making is perceived by patients compared to traditional general practitioner (GP)-directed approaches in managing MLTC. By exploring attitudes and barriers to AI implementation, the study aims to foster a comprehensive understanding of the landscape surrounding patient views on AI-enabled decision-making tools, particularly within the primary care context.

## Methods

### Study Design

Data were gathered using semistructured interviews conducted by telephone or via video link (Zoom or Microsoft Teams) between July and November 2023, in line with our published protocol [12,15]. The interview topic guide was developed by the multidisciplinary research team, including members of a patient advisory group (PAG), clinicians, a medical sociologist, qualitative researchers, and other researchers, such as health economists and health data scientists. It was informed by a review of the published literature (unpublished) about the acceptability and utility of using AI for decision-making in health care. Interviews were designed to last for around 30 to 40 minutes.

The interview topic guide, piloted with PAG members and modified based on their feedback, comprised open-ended questions and prompts to elicit detailed responses, with questions focusing on participants’ perceptions of the advantages and disadvantages of the use of AI-informed clinical decision-making. The interview topic guide for participants is provided in Multimedia Appendix 1. Prior to the interview, participants were provided with a 1-page briefing note on AI. The briefing note, also developed with the PAG, explained what AI is and how it can be used in health care. At the start of the interview, the interviewer also provided further explanation and answered any participant questions about AI.

### Participants and Recruitment

Participants were recruited using purposive sampling via 2 pathways. The first recruitment method used the Clinical Practice Research Datalink (CPRD) Aurum database, an anonymized, population-based, UK database of electronic primary care records [16]. CPRD contains validated clinical and demographic data representative of the UK population [17]. These data, including diagnoses, tests, and prescriptions, were used to identify potential participants [18]. The inclusion criteria include patients who were aged 18 years and above and patients with 2 or more long-term health conditions. The exclusion criteria include patients who were diagnosed with a terminal illness (prognosis of 12 mo or less) and patients who may lack capacity to provide informed consent or undertake study activities.

Using the CPRD patient referral service, invitations were sent via GP practices in the West Midlands, prioritizing more deprived areas with higher proportions of minoritized ethnic groups and patients with MLTC. The Data Extraction for Epidemiological Research (DExtER; developed by researchers at the University of Birmingham) software was used by the research team to generate a pool of potentially eligible patients [19]. Eligible patients identified via CPRD were sent an invitation letter and participant information sheet (PIS) by their GP practice (Multimedia Appendix 2).

**Figure 1.**
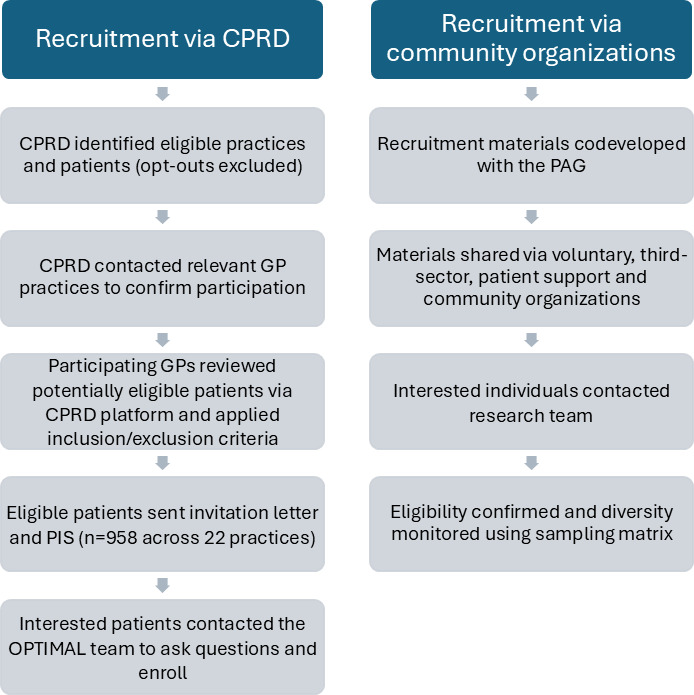
OPTIMAL study recruitment diagram. CPRD: Clinical Practice Research Datalink; GP: general practitioner; OPTIMAL: Optimizing Therapies, Disease Trajectories, and Artificial Intelligence-Assisted Clinical Management for Patients Living With Complex Multimorbidity; PAG: patient advisory group; PIS: participant information sheet.

Fewer patients than expected passed GP screening (56%), and the response rate among those invited was low. To augment recruitment, ethical approval was obtained to implement an additional recruitment pathway. A community-based approach was introduced, with recruitment materials (Multimedia Appendix 3) circulated through voluntary, third-sector patient support and community organizations. The recruitment strategy for both pathways is shown in Figure 1.

Interviews were conducted until data saturation was reached and no new themes were identified [20]. A sampling matrix was used to monitor participant demographics during recruitment to support diversity in age, sex, ethnicity, and number of long-term conditions [21]. In total, 21 participants were recruited via CPRD, and 8 participants were recruited from the community pathway.

### Patient and Public Involvement

Members of the public and patients with MLTC have been involved in every stage of the OPTIMAL study. A PAG was established at study inception, comprising 9 members who have lived experience of MLTC (either directly or as a carer). PAG recruitment considered diversity in terms of personal characteristics and experience of MLTC. PAG members were involved in codeveloping the topic guide and participant-facing documents (PIS, invitation letter, and consent form) by reviewing and commenting on each iteration. Quarterly PAG meetings supported the qualitative study by reviewing findings and ensuring that the study aims remained meaningful to patients with MLTC.

### Procedure and Data Analysis

All interviews were conducted by SF, a Research Fellow at the University of Birmingham, working on qualitative studies based within health research. The interviews were audio-recorded and transcribed verbatim by a transcription company contracted to the University of Birmingham. All transcripts were anonymized and stored on a secure, password-protected server that only authorized personnel had access to, in accordance with UK data protection regulations and in line with University of Birmingham data protection and data security policies.

Personal identifying information was removed from transcripts prior to analysis. Transcripts were analyzed thematically [22]. Following familiarization, codes were assigned using an inductive approach [23]. A sample of transcripts was independently coded by JC (a Clinical Research Fellow with experience in qualitative research) to ensure consistency. After the coding process, the codes were merged into initial themes, which were then further refined by 2 researchers (CS and LJ).

During data coding and theme development, the findings were discussed with the PAG and members of the multidisciplinary research team to identify any areas for further exploration or clarification in subsequent interviews. Coding differences were worked through in a discussion-based, collaborative process with the whole team, which helped maintain clarity and consistency while recognizing the reflexive nature of qualitative analysis. Microsoft Word and Excel (Microsoft 365) were used to read the transcripts and to support coding and theme development.

The COREQ (Consolidated Criteria for Reporting Qualitative Research) checklist was completed to support transparent reporting. The checklist is provided in Checklist 1, and the corresponding reflexivity statement is provided in Multimedia Appendix 4.

### Ethical Considerations

Ethical approval for the study was obtained from the National Health Service (NHS) Research Ethics Committees (reference 22/SC/0210). Ethical approval to use the CPRD dataset to select the eligible practices and potentially eligible patients was obtained from the CPRD Research Data Governance Expert Review Committee and the Central Advisory Committee (reference 21_000683). Electronically completed written consent or audio informed consent was collected from participants, and they were informed of their right to withdraw from the study at any time. As part of the consent process, participants were asked for permission to be contacted at a later date with a view to inviting them to take part in a follow-up interview related to a separate theme within the study. Participants’ privacy and confidentiality were maintained throughout the study. Interviews were anonymized, and all data were stored securely at the University of Birmingham. A £15 (US $20.17) shopping voucher was offered to participants for their contribution to the study.

## Results

### Participant Characteristics

Recruitment continued until data saturation was achieved. In total, 29 interviews were conducted with participants of different sex, ages, and ethnic backgrounds (Table 1). The sample was predominantly female and largely White British, with smaller representation from Asian/British Asian and mixed ethnic backgrounds. Participant age range spanned from 20 to above 80 years, with the majority (17/29, 58%) aged 70 years and above.

**Table 1.** Characteristics of the participants (N=29).

Characteristic and subgroup	Values, n (%)
Sex	
Female	18 (62)
Male	11 (38)
Age range (y)	
20‐29	1 (3.45)
40‐49	2 (6.90)
50‐59	4 (13.79)
60‐69	5 (17.24)
70‐79	11 (37.93)
≥80	6 (20.69)
Ethnicity	
White British	24 (82.75)
Asian/British Asian	4 (13.79)
White and Black African	1 (3.44)
Number of long-term conditions	
2-3	12 (41.39)
4 or more	17 (58.6)
Total	29 (100)

### Main Themes

Overall, the interviews found 2 broad themes: potential benefits and potential risks of AI (Table 2). Potential benefits included 4 subthemes, such as enhanced efficiency and accuracy, improved access to information for patients and clinicians, potential early detection of changes in health state, and reducing health care inequalities. Five subthemes were identified for the potential risks of AI, such as the loss of human touch, privacy and data security, transparency of the AI tool and associated information, trust in and accuracy of AI tools, and potential bias and inequality.

**Table 2.** Summary of themes and subthemes identified in relation to artificial intelligence (AI) use in multiple long-term conditions (MLTC) care.

Themes and subthemes	Definition
Potential benefits of AI for MLTC care	
Enhanced efficiency and accuracy in processing medical information	The ability of AI to quickly analyze large volumes of clinical data, reduce errors, and support more accurate and efficient decision-making in complex care
Improved access to information for patients and clinicians	AI providing clearer, more accessible health information, helping both patients and clinicians understand conditions, treatment options, and up-to-date evidence
Potential for early detection of changes in the health state	AI identifying subtle patterns or early signs of deterioration, enabling preventative action or timely intervention
Reducing health care inequalities	AI supporting more consistent care across patient groups by standardizing information, highlighting overlooked needs, and improving access for those with lower health literacy
Potential risks of AI for MLTC care	
Loss of human touch	Concerns that AI may reduce personal interaction, empathy, and the relational aspects of care that patients value.
Privacy and data security	Worries about how personal health data are stored, shared, or accessed within AI systems and risks of unauthorized use
Lack of transparency	Uncertainty about how AI reaches decisions, how algorithms are developed, what influences their recommendations, and how decisions are made within the health system about AI tools
Trust and accuracy and AI outputs	Need for reassurance that AI recommendations are reliable, safe, and checked by clinicians, with fears about overreliance or errors
Potential bias and inequality	Concerns that AI trained on nonrepresentative data could reinforce existing disparities or produce less accurate outputs for underrepresented groups

### Potential Benefits of AI

#### Enhanced Efficiency and Accuracy in Processing Medical Information

Participants commonly described the potential for AI to improve the efficiency of consultations and aid in joint decision-making. It was acknowledged that AI could help process large amounts of medical information more quickly than is currently possible, especially in relation to complex medication regimens. Several participants reflected on past experiences where medication interactions had been overlooked and suggested that AI could reduce this risk while also saving time for both patients and clinicians.

*Well I have felt that at times, medication has been prescribed without regard to what I am already taking, […] but AI could do it so much faster and would take away the need for me to do it myself.*

[ID3, female, 75 years old]

Beyond medication management, participants also viewed AI as a tool that could support clinical decision-making during consultations by rapidly analyzing information and presenting relevant options. The predictive capability of AI in identifying potential future health conditions was viewed as valuable. Participants noted that such tools could improve patient care while saving time for clinicians and reducing costs for the NHS.

*Maybe it would save them time, money, resources. I think it would probably be a really good tool for them as well because they can help their patients better…*

[ID16, female, 45 years old]

#### Improved Access to Information for Patients and Clinicians

Participants appreciated that AI had the potential to provide more accessible information about their conditions, supporting greater understanding and involvement in managing their health. In particular, AI was seen as a way of offering patients additional information about alternative options that they might benefit from. One participant described feeling powerless in managing a complex condition and saw AI as a way to gain clearer information.

*Because I think the NHS is under resourced and they don’t have time to explain all the different things to you […] I don’t feel like I’m proactively doing much at the moment apart from taking my drugs. So, I feel very powerless and I would like more.*

[ID22, female, 28 years old]

Participants also recognized that AI could support clinicians in helping patients manage MLTC, by drawing attention to new or previously overlooked information and improving awareness of emerging evidence. In addition, AI was seen as having the potential to improve access to information for clinicians by consolidating and analyzing complex medication data.

*Well, you’d most probably get the doctors to understand a bit more. If it brings up new things and the doctor decides to think about that as well, all the better for you know, getting this artificial intelligence to everyone you know.*

[ID24, male, 57 years old]

#### Potential for Early Detection of Changes in Health State

AI was perceived as potentially capable of detecting subtle changes or patterns indicative of disease progression earlier than traditional methods, allowing for timely interventions. Participants described how AI could act as a “comfort blanket,” offering reassurance that health changes would be monitored and addressed promptly.

*But if I’m moving along the pathway, the computer says I need to do a blood test, I’m sending you for a blood test, the computer says we need to do this test just to monitor you for this – I feel it’s a comfort blanket.*

[ID13, female, 65 years old]

There was a sense that AI may be able to provide indications for preventative measures for presenting conditions.

*I think that would be a good thing, because prevention is better than cure isn’t it really. So, if you’re prone to it then you can make the changes before it actually comes to the seriousness of the complaint.*

[ID8, female, 82 years old]

#### Reducing Health Care Inequalities

Some participants believed that AI could help reduce health care disparities by standardizing care practices and ensuring equitable access to high-quality health care services. One participant suggested that AI may be especially useful for patients who may be less “health literate.”

*But I think in cases of some individuals who may not have insight, and they have complex medical conditions, I think AI would be a great, great help to them I think and to the practitioners who are looking after [them].*

[ID5, male, 71 years old]

### Potential Risks of AI

#### Loss of Human Touch

Participants expressed concerns that the use of AI could reduce the human elements of care (eg, communication and compassion). In particular, there were worries that consultations might become more transactional, with clinicians relying on automated outputs rather than engaging fully with the individual patient. As 1 participant explained:

*I would hope that it wouldn’t become sort of rote in so far that the computer spills out information and the doctor immediately doesn’t really look at it, doesn’t really pay attention to it and then just hands over a prescription.*

[ID3, female, 75 years old]

Furthermore, some participants expressed anxiety that AI might eventually replace face-to-face interactions with their GP, highlighting the importance of being able to have a dialogue with health care professionals. There was a sense that this may more adversely impact older people.

*I think it’s contact, isn’t it? The perception is that you won’t see anyone, it’ll just be a computer with a face and you deal with it. I still think from, certainly from an older person’s perspective, you want to sit down and talk to someone.*

[ID26, male, 76 years old]

#### Privacy and Data Security

Participants raised concerns about the privacy and security of their health information when handled by AI systems. There were worries that personal data could be accessed by unauthorized individuals, highlighting the importance of strong safeguards and clear data protection measures when implementing AI in health care.

*Well, I suppose people worried about their information being available because other people could get access to it...*

[ID29, male, 82 years old]

#### Lack of Transparency

Concerns were also expressed about the transparency of AI and how AI-driven decisions are made. Participants indicated a desire for reassurance that AI tools would be thoroughly checked and validated, while acknowledging that some aspects of clinical decision-making are already not fully visible to patients. This was demonstrated by 1 participant who stated they would like reassurance that AI tools are carefully checked but, at the same time, highlighted a lack of perceived transparency currently with clinician-based decision-making.

*As long as the software is checked and simulated, I’m good with that. I can’t see the software but I’m pretty sure that there’s something in there that they’re saying “Try this. Try that. Do this. Do that.” So, we’re not far away now are we, the way they operate?*

[ID26, male, 76 years old]

Some participants also questioned whether financial or organizational incentives could influence how AI is developed and implemented within health care. They questioned whether AI would truly serve patients’ best interests or be driven by profit and efficiency within the health care system, which further highlights the need for transparency in how decisions about the introduction of AI are made.

*When everything is about money and profit these days, you have to question whether is AI being proposed as a solution for patients or a solution for cheaper private healthcare?*

[ID19, male, 64 years old]

#### Trust and Accuracy of the AI Outputs

Participants emphasized the need for reassurance about the accuracy and reliability of AI-generated outputs. They suggested that AI should support, rather than replace, clinical judgment with safeguards, such as human oversight in place.

*I think there needs to be some sort of second opinion thing built into it because AI is not foolproof. So, there’s definitely some sort of second opinion.*

[ID21, male, 65 years old]

Trust in AI also appeared to vary by age, with some participants suggesting that younger people might be more comfortable relying on digital technologies than older individuals, who may feel more cautious or uncertain. There was also a sense of caution about overreliance on AI, with concerns that algorithms may not capture individual variations or predict all outcomes accurately.

*I’d like to know first of all, is it going to be beneficial to me in the long run. It’s all right saying “We can put you on this medication” but you know, it can’t really predict side effects and those side of things.*

[ID1, female, 41 years old]

#### Potential Bias and Inequality

Participants expressed concern that AI could reinforce existing inequalities in health care if the data used to train the AI do not adequately represent diverse populations. There was particular awareness that individuals from minority ethnic, cultural, or neurodiverse backgrounds may be misrepresented or overlooked, leading to less accurate or appropriate recommendations for these groups. As 1 participant explained, this would potentially lead to AI making recommendations informed by data that inadequately reflect the circumstances of many people, particularly those from underrepresented or disadvantaged communities.

*When it comes to it, I think it can be really good and a really useful tool, as long as you’ve got the right information. I find a lot of the time—and this is speaking from lived experience—within south Asian communities and with Black communities—people do not talk about their physical or mental health. So AI will only know about white communities who talk about these conditions.*

[ID16, female, 45 years old]

## Discussion

### Principal Findings

This study found that participants had mixed views regarding the use of AI to inform joint decision-making for patients with complex MLTC in a primary care setting. Most recognized potential benefits included improved efficiency and accuracy, enhanced access to information, the ability to detect early changes in health status, and the potential to reduce inequalities. However, nearly all participants also highlighted concerns, particularly around the loss of human touch, data security, transparency, accuracy, and potential biases within AI.

### Comparison With Other Work

These findings are consistent with existing literature reporting mixed patient views on AI in health care [24,25]. Previous research has shown that patients view AI in health care with cautious optimism, recognizing its potential to improve diagnosis, efficiency, and care quality while emphasizing the need for human oversight and transparency in decision-making [26]. In this study, participants identified improved access to information as a potential benefit, including greater access to information about specific conditions and potential contraindications or medication side effects. Similar findings have been reported elsewhere, including research on AI chatbots for older adults, which identified improved access to easy-to-understand medication and adverse reaction information as a key benefit [27]. However, prior work has also emphasized that patients require a certain level of health literacy to fully benefit from such technologies [28].

Our findings presented here also highlight the potential risks. Particular concerns persist around the use of AI, in terms of data quality, bias, privacy, and the risk of diminishing human interaction and patient autonomy [26]. In this study, privacy and data security were raised by a small number of participants, although not universally. Other research has documented patient concerns regarding privacy and data security risks [29,30]. Bias in AI may arise from the sources of data or existing inequities embedded in datasets [31,32], including evidence of bias in AI risk prediction tools that led to inequitable allocation of health care resources for Black patients [33]. These findings highlight the need to address bias in AI design and implementation. Patients with MLTC in primary care expressed concerns and expectations similar to those reported in other health care settings.

Participants also expressed concern about the potential loss of human touch in health care delivery. Many valued the time spent with their GP and worried that AI might make care less personalized. This aligns with other studies [29,30] and may reflect some misunderstandings about different ways in which AI could be used within GP consultations to support personalized recommendations. This gap in understanding reflects broader evidence of limited public awareness of AI and its potential applications in health care [26,34].

This study adds the perspectives of those living with MLTC, a group that has traditionally been underrepresented in discussions around AI and digital health [35]. In addition, the study also focuses on primary care, a setting with enormous perceived potential, but where the use of AI tools has been more limited [36]. Importantly, the findings reflect the UK primary care context, highlighting issues such as GP consultation structure, patient-provider relationships, and NHS-specific resource constraints that may influence AI adoption and implementation.

There are a number of implications for policy and practice. First, AI development should involve a diverse range of patients, particularly those with lived experience of complex MLTC, who can provide unique perspectives [37]. There are also implications around the need for AI tools to enhance patient-clinician decision-making, while also recognizing the importance and value of human interaction [29]. This highlights the importance of building trust into the design and implementation of AI, particularly by supporting patient health literacy and providing guided access to relevant and authoritative tools [28]. Integration within GP consultations may help clinicians interpret AI outputs, reinforcing trust and joint decision-making.

Concerns around privacy, security, and transparency remain important, but AI also has the potential to increase accuracy and personalization, particularly for MLTC, where holistic approaches to care are needed [38]. Further consultation and reassurance are needed for the public to trust that their data are being used appropriately by AI tools. Clear governance arrangements and robust data protection safeguards are required to manage how patient data are collected, stored, and processed, including ensuring compliance with legislation, such as the General Data Protection Regulation.

Participants provided important insights into how current care pathways and research often fail to adequately represent diverse populations, including different ages, ethnicities, migrant status, neurodiversity, disability, mental health conditions, or MLTC. This research further highlights the need for strengthened efforts to address potential biases and inequalities, using collaborative and multifaceted approaches that engage minority and marginalized communities to assess whether AI tools meet their needs or risk exacerbating disparities [39].

### Strengths and Limitations

This study has several strengths. The study used a broad recruitment strategy through the CPRD database search and outreach through community, patient, and third-sector organizations. There was a robust approach to data collection and analysis, with the involvement of a multidisciplinary research team. Patient and public involvement was embedded throughout the study, with insights informing study design, recruitment, and analysis. This enhanced the credibility and applicability of findings by ensuring that their perspectives shaped study design, interpretation, and implementation [40].

There were several limitations to the research. The low response rate may reflect a lack of interest in or confidence discussing AI-related topics among the wider public. As noted elsewhere, individuals without direct experience of AI may find it difficult to engage with such topics, particularly when examples of AI applications are not clearly defined [41]. It is also likely that those who chose to participate had a particular interest in the subject, meaning the sample may not be fully representative of the wider patient group with MLTC. In addition, although there was some diversity in the participants interviewed, there were fewer young people, men, and people from ethnic minorities. There might have been some views and perspectives that were not included in this analysis, which is important given the potential implications of biases in AI [31]. More research is needed with different demographics, for example, young people and ethnic minorities, where perceptions of AI may vary. Furthermore, the lower-than-expected response to the research may reflect the demands of a 60-minute interview, with potential for a further interview later. Patient burden should be considered for future studies.

Although ethical approval was obtained, ethical considerations were also embedded within the study design. Prior to the interviews, participants received information about AI, and there was also information within the PISs. This may have framed participants’ perceptions about AI. For example, we did not specifically raise ethical issues around the use of AI in this information. As this is an area of rapid change, people’s views and perceptions may change over time, but exploring this was beyond the scope of the study. A more in-depth exploration of ethical concerns related to AI implementation warrants future research.

### Conclusion

This study provides novel findings into patient perspectives on the use of AI to support primary care consultations for individuals living with MLTC. Participants identified several potential benefits, including greater efficiency and improved accuracy and greater access to information. However, they also expressed concerns about potential risks, such as reduced human interaction, data privacy, and the accuracy of AI and its potential to exacerbate inequalities. The findings highlight the need for transparency in AI data use and clear communication and public engagement to build understanding and trust in AI use within health care. Further research is needed to explore how AI might influence clinical interactions in primary care and patient confidence in these tools over time, including longitudinal studies to examine how perceptions change with increased exposure and use. Future work should also prioritize the inclusion of more diverse populations to better understand how experiences and the impact of AI may differ across demographic and socioeconomic groups.

## Supplementary material

10.2196/87507 Multimedia Appendix 1 Interview topic guide.

10.2196/87507 Multimedia Appendix 2 Participant information sheet.

10.2196/87507 Multimedia Appendix 3 Community recruitment poster.

10.2196/87507 Multimedia Appendix 4 Reflexivity statement.

10.2196/87507 Checklist 1 COREQ checklist.

## Abbreviations

AI: artificial intelligence
CPRD: Clinical Practice Research Datalink
DExtER: Data Extraction for Epidemiological Research
GP: general practitioner
MLTC: multiple long-term conditions
NHS: National Health Service
OPTIMAL: Optimizing Therapies, Disease Trajectories, and Artificial Intelligence-Assisted Clinical Management for Patients Living With Complex Multimorbidity
PAG: patient advisory group
PIS: participant information sheet

## References

[1] Chowdhury SR, Chandra Das D, Sunna TC, Beyene J, Hossain A (2023). Global and regional prevalence of multimorbidity in the adult population in community settings: a systematic review and meta-analysis. EClinicalMedicine.

[2] Valabhji J, Barron E, Pratt A (2024). Prevalence of multiple long-term conditions (multimorbidity) in England: a whole population study of over 60 million people. J R Soc Med.

[3] Pati S, MacRae C, Henderson D, Weller D, Guthrie B, Mercer S (2023). Defining and measuring complex multimorbidity: a critical analysis. Br J Gen Pract.

[4] Baker JM, Grant RW, Gopalan A (2018). A systematic review of care management interventions targeting multimorbidity and high care utilization. BMC Health Serv Res.

[5] Soley-Bori M, Ashworth M, Bisquera A (2021). Impact of multimorbidity on healthcare costs and utilisation: a systematic review of the UK literature. Br J Gen Pract.

[6] Larkin J, Foley L, Smith SM, Harrington P, Clyne B (2021). The experience of financial burden for people with multimorbidity: a systematic review of qualitative research. Health Expect.

[7] Hanlon P, Hannigan L, Rodriguez-Perez J (2019). Representation of people with comorbidity and multimorbidity in clinical trials of novel drug therapies: an individual-level participant data analysis. BMC Med.

[8] Ng DTK, Leung JKL, Chu KWS, Qiao MS (2021). AI literacy: definition, teaching, evaluation and ethical issues. Proc Assoc Inf Sci Technol.

[9] Arora A, Alderman JE, Palmer J (2023). The value of standards for health datasets in artificial intelligence-based applications. Nat Med.

[10] Khosravi M, Zare Z, Mojtabaeian SM, Izadi R (2024). Artificial intelligence and decision-making in healthcare: a thematic analysis of a systematic review of reviews. Health Serv Res Manag Epidemiol.

[11] Kueper JK, Terry AL, Zwarenstein M, Lizotte DJ (2020). Artificial intelligence and primary care research: a scoping review. Ann Fam Med.

[12] Gunathilaka NJ, Gooden TE, Cooper J (2024). Perceptions on artificial intelligence-based decision-making for coexisting multiple long-term health conditions: protocol for a qualitative study with patients and healthcare professionals. BMJ Open.

[13] Khullar D, Casalino LP, Qian Y, Lu Y, Krumholz HM, Aneja S (2022). Perspectives of patients about artificial intelligence in health care. JAMA Netw Open.

[14] Moy S, Irannejad M, Manning SJ (2024). Patient perspectives on the use of artificial intelligence in health care: a scoping review. J Patient Cent Res Rev.

[15] Archibald MM, Ambagtsheer RC, Casey MG, Lawless M (2019). Using Zoom videoconferencing for qualitative data collection: perceptions and experiences of researchers and participants. Int J Qual Methods.

[16] Wolf A, Dedman D, Campbell J (2019). Data resource profile: Clinical Practice Research Datalink (CPRD) Aurum. Int J Epidemiol.

[17] Shiekh SI, Harley M, Ghosh RE (2023). Completeness, agreement, and representativeness of ethnicity recording in the United Kingdom’s Clinical Practice Research Datalink (CPRD) and linked Hospital Episode Statistics (HES). Popul Health Metr.

[18] CPRD reach. CPRD (Clinical Practice Research Datalink).

[19] Gokhale KM, Chandan JS, Toulis K, Gkoutos G, Tino P, Nirantharakumar K (2021). Data extraction for epidemiological research (DExtER): a novel tool for automated clinical epidemiology studies. Eur J Epidemiol.

[20] Rahimi S, Khatooni M (2024). Saturation in qualitative research: an evolutionary concept analysis. Int J Nurs Stud Adv.

[21] Negrin KA, Slaughter SE, Dahlke S, Olson J (2022). Successful recruitment to qualitative research: a critical reflection. Int J Qual Methods.

[22] Braun V, Clarke V (2019). Reflecting on reflexive thematic analysis. Qual Res Sport Exerc Health.

[23] Saldaña J (2024). Expanding Approaches to Thematic Analysis.

[24] Witkowski K, Dougherty RB, Neely SR (2024). Public perceptions of artificial intelligence in healthcare: ethical concerns and opportunities for patient-centered care. BMC Med Ethics.

[25] Gundlack J, Thiel C, Negash S (2025). Patients’ perceptions of artificial intelligence acceptance, challenges, and use in medical care: qualitative study. J Med Internet Res.

[26] Richardson JP, Smith C, Curtis S (2021). Patient apprehensions about the use of artificial intelligence in healthcare. NPJ Digit Med.

[27] Gudala M, Ross MET, Mogalla S, Lyons M, Ramaswamy P, Roberts K (2022). Benefits of, barriers to, and needs for an artificial intelligence-powered medication information voice chatbot for older adults: interview study with geriatrics experts. JMIR Aging.

[28] Schulz PJ, Nakamoto K (2013). Patient behavior and the benefits of artificial intelligence: the perils of “dangerous” literacy and illusory patient empowerment. Patient Educ Couns.

[29] Young AT, Amara D, Bhattacharya A, Wei ML (2021). Patient and general public attitudes towards clinical artificial intelligence: a mixed methods systematic review. Lancet Digit Health.

[30] Nelson CA, Pérez-Chada LM, Creadore A (2020). Patient perspectives on the use of artificial intelligence for skin cancer screening: a qualitative study. JAMA Dermatol.

[31] Gichoya JW, Thomas K, Celi LA (2023). AI pitfalls and what not to do: mitigating bias in AI. Br J Radiol.

[32] Agarwal R, Bjarnadottir M, Rhue L (2023). Addressing algorithmic bias and the perpetuation of health inequities: an AI bias aware framework. Health Policy Technol.

[33] Obermeyer Z, Powers B, Vogeli C, Mullainathan S (2019). Dissecting racial bias in an algorithm used to manage the health of populations. Science.

[34] Vo V, Chen G, Aquino YSJ, Carter SM, Do QN, Woode ME (2023). Multi-stakeholder preferences for the use of artificial intelligence in healthcare: a systematic review and thematic analysis. Soc Sci Med.

[35] Yi M, Hui Y, Hu L, Zhang W, Wang Z (2024). The experiences and perceptions of older adults with multimorbidity toward e-health care: a qualitative evidence synthesis. Telemed J E Health.

[36] Katonai G, Arvai N, Mesko B (2025). AI and primary care: scoping review. J Med Internet Res.

[37] Badawy W, Zinhom H, Shaban M (2025). Navigating ethical considerations in the use of artificial intelligence for patient care: a systematic review. Int Nurs Rev.

[38] Koirala B, Peeler A, Dennison Himmelfarb C, Davidson PM (2023). Living with multiple chronic conditions: how we achieve holistic care and optimize health outcomes. J Adv Nurs.

[39] Wang T, Emami E, Jafarpour D, Tolentino R, Gore G, Rahimi SA (2025). Integrating equity, diversity, and inclusion throughout the lifecycle of artificial intelligence for healthcare: a scoping review. PLOS Digit Health.

[40] Rahman A, Nawaz S, Khan E, Islam S (2022). Nothing about us, without us: is for us. Res Involv Engagem.

[41] Scott IA, Carter SM, Coiera E (2021). Exploring stakeholder attitudes towards AI in clinical practice. BMJ Health Care Inform.

