# Supplementary material for: Exploring Patient Perspectives on the Use of Artificial Intelligence to Inform Joint Decision-Making for Patients With Multiple Conditions in Primary Care in the United Kingdom: Qualitative Study
Source: J Med Internet Res. 2026 Apr 21;28:e87507. doi: 10.2196/87507 (PMC13099014; doi:10.2196/87507)
Supplement: Multimedia Appendix 1 [file jmir-v28-e87507-s001.docx]

**OPTIMIsing therapies, disease trajectories, and AI assisted clinical management for patients Living with complex multimorbidity (OPTIMAL):**

**Interview guide: Patient Interview**

**This is the starting topic guide. The overarching objectives will remain the same, but questions and prompts will be developed as interviews are undertaken to incorporate any important themes emerging from the interviews.**

**Before the interview begins**

- Ensure the participant has read the information leaflet
- Ensure the participant has had the opportunity to ask any questions about the research including issues about confidentiality, the findings of the research and where the research will be disseminated before being asked to agree to each item on the consent form.
- Start audio-recording
- Go through each item on the consent form and record their verbal consent. Explain that you will send/email a copy of the consent form for their records. They should already have a copy of the participant information sheet with details about the study, how to withdraw etc
- Explain that they don’t have to answer all the questions just because they have consented to the interview, and that they can take a break or stop the interview at any time.
- Explain that you are there to understand more about their experiences and that they will have some time at the end of the interview to talk about any other issues that are important to them that may not have been covered by the questions.
- Check that they are happy to continue to be audio-recorded.
- Begin the interview.

**TOPICS TO BE COVERED IN THE INTERVIEW**

Patient’s reflections on living with/managing long-term conditions

**Could you tell me about your health conditions?** Prompts – how long have you had conditions, how have they developed over time

**What are your experiences of managing patients your long-term conditions?** Prompts – medication, self-management, navigating primary/specialist care, relationships with clinicians/HCPs

**Have you experienced any difficulties managing your conditions?** Prompts -

- Side-effects from medication (impact on appetite, understanding which medications are for which condition; remembering to take medication)
- Multiple appointments for different conditions; opportunities to see GP/specialists; information sharing;
- Impact of conditions upon other activities e.g. work, time to socialise, leisure activities, access
- Impact upon relationships

 Acceptability of AI in clinical decision making

**Attitudes and understanding about AI in general terms** (briefing guide of discussion points about AI)

Have you any thoughts about AI and how it is used in everyday activities?

**Do you have any experience of AI being used to manage your healthcare?**

Attitudes towards and perspectives about AI health care in managing long term conditions

In this study interview we want to explore the advantages and limitations of AI directed clinical decision making and compare this with doctors making decisions.

**How do you feel about how clinical decisions are made about your healthcare? E.g. do you feel involved in decisions?**

**Do you think the use of AI (computer programs) in healthcare decision making could be beneficial? If so, how could it be used? If not, what are the reasons why?**

**Do you think that AI (computer programs) could improve clinical decision making?**

**Do you think that AI (computer programs) could help you manage your health conditions? If so, how may this benefit you and what would ‘good’ look like?**

**In which situations do you think AI (computer programs) should not be used?**

**What would your concerns be, if any?**

**How do you feel that using AI (computer programs) for clinical decision making may compare to clinician/patient making decisions about your health?**

**Can you think of ways AI (computer programs) making decisions about your care may be preferable to clinicians making decisions about your care?**

**Can you think of ways clinician/patient guided decisions about your care are preferable to AI directed decisions about your care? E.g. continuity of relationship; importance of relationship with HCP**

**What kind of questions might you want to ask before agreeing to treatment that was directed by AI?**

**What kind of things may influence your choice between AI or clinician/patient guided decisions about your care?**

**How confident would you feel if prescription decisions were directed by AI (computer program/predictive algorithm) rather than your doctor?**

Very comfortable   Mostly comfortable  Not sure    Mostly uncomfortable  Very uncomfortable

**Why?**

Stage two: Vignettes – validity of AI in clinical decision making

We are developing a tool that will detect what other conditions a patient may develop in the future based upon their current health conditions. It will also provide information about what sort of medications should be prescribed (to achieve maximum health benefits and reduce side-effects etc)

*Participants will have been sent a copy of the case vignette prior to the interview*

We will present to the participant, a simulated patients disease clusters, to compare how aspects of the AI patient care management fit in with their own experiences:

The case vignette will present a simulated patients disease clusters, with 4 health conditions showing:

- How conditions developed
- What other conditions the patient developed
- Impacts of conditions upon their lifestyle and relationships etc
- How medications were managed
- How overall health management is navigated via the AI algorithm

**How does the patient’s experience of being diagnosed with multiple health conditions compare with your experience? Explain some of the similarities and the differences.**

**How does the patient’s experience of feeling that some of the worsening of their symptoms and being diagnosed with other health conditions are due to the medicines he is taking compare with your experience? Explain some of the similarities and the differences.**

**What other things would you want to know about this patients to inform decisions about management of their treatment e.g lifestyle factors?**

**Would it be useful to know for example what conditions may develop in the future?**

**Would this impact upon things like lifestyle changes? E.g. dietary/exercise/self-monitoring regime**

**How would you feel about a computer programme predicting the medications you may benefit most from rather than your doctor?**

Very comfortable     Mostly comfortable      Not sure      Mostly uncomfortable      Very uncomfortable

**Does this case simulation reflect your own kind of experiences and preferences in terms of how your cMM are managed?**

**What do you think are the most important things that researchers need to consider when developing computer programmes to manage patient care?**

Conclusion of interview

Now we’ve talked a bit about AI could be used, how do you feel about it?

Thinking back to the difficulties you talked about in managing treatments, do you think AI could have an effect on this?

Thank you. That was my last question.

Is there anything you would like to add about the things we talked about but have not covered in the interview?

Any questions from interviewee

Reminder of study contact details and signposting
