# Supplementary material for: Exploring Patient Perspectives on the Use of Artificial Intelligence to Inform Joint Decision-Making for Patients With Multiple Conditions in Primary Care in the United Kingdom: Qualitative Study
Source: J Med Internet Res. 2026 Apr 21;28:e87507. doi: 10.2196/87507 (PMC13099014; doi:10.2196/87507)
Supplement: Multimedia Appendix 2 [file jmir-v28-e87507-s002.docx]

**
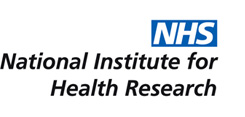

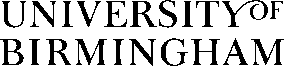
**

**OPTIMIsing therapies, disease trajectories, and AI assisted clinical management for patients Living with complex multimorbidity (OPTIMAL)**

**Using Artificial Intelligence (AI) to help people living with four or more medical conditions to receive the best treatment and care**

**Participant information sheet: patient interviews**

**Introduction**

We would like to invite you to take part in a research study. Before you decide to take part, we would like to explain why we are doing this study and what your involvement will mean for you. Please take time to read this information sheet and discuss it with others if you wish. Ask us if there is anything that is not clear or if you would like more information before deciding whether or not to take part. Our contact details are at the end of this information sheet.

**What is the study about?**

The study aims to use artificial intelligence (AI) to produce computer programmes and tools that will help improve the treatment and choice of medications in patients with clusters of multiple long-term conditions (we say these people have complex multimorbidity or cMM).

Artificial intelligence (or AI) is a computer system that can conduct tasks that would normally need human intelligence. Newer AI methods makes it easier to process large amount of health data in a short time. These AI methods can give doctors and patients information that may help improve the care of people with multiple long-term health conditions

AI can be used in healthcare to help guide the diagnosis of long-term health conditions, plan the best treatment strategies, and predict the next health condition that people might develop. This is especially relevant for people who have several different health conditions because the guidelines that doctors and healthcare professionals may use for one condition does not routinely consider other health conditions.

AI-based decision-making tools (computer programs) for managing multiple health conditions may help patients and healthcare professionals make more well-informed shared decisions. Patients and healthcare professionals may see many benefits to the use of AI in healthcare. However, they may be concerned this could be harmful or that it could affect the relationships between healthcare professionals and patients. Also, it is important that in practice these AI-based decision-making tools are presented in a way that helps guide decision-making for both healthcare professionals and patients and considers factors in these tools are important to them.

Data collected from this study will give new insights to how healthcare professionals and people with multiple long-term health conditions view AI-based decision-making tools. It will also tell us which factors in the computer program are important to them and what options they prefer. This information will also be used to help to develop how these tools are used in clinical practice.

**How will we do this?**

1. By using artificial intelligence (AI) methods with electronic health records to generate data models that tell us how the different mixes of conditions arise over time and how certain drugs can make this better or worse.
2. By asking people with cMM and doctors about their knowledge and views about using AI to make decisions about health care.
3. By using AI computer techniques to combine data, and together with the input from people with cMM and doctors, develop a computer program. This will predict which drug we should give and when we should give it to someone with cMM to reduce risk and bring about maximum benefit. It will also tell us what disease people may get next. These predictions will be based upon data gathered from a large number of patients who have similar conditions and prescribed medication.
4. By examining the best way to present information in the AI tool to people with cMM and doctors by asking them about what is important to them and what options they prefer.

Our team includes patients, public members, and world leading experts from universities with expertise in biology, AI, medicine, health service research, public health, and general practice. Working as a multidisciplinary team we hope to improve the health and care of people with cMM.

**Why have I been chosen?**

You have been invited because we would like to talk to you about your experiences living with four or more long-term health conditions.

**Do I have to take part?**

The decision about whether you choose to take part is up to you. Whether you decide to take part or not will have no effect upon your healthcare and treatment and you can leave the study at any time, without giving a reason.

**What will happen to me if I take part?**

If you choose to take part, you can contact the research team by telephone or email (details below). The researcher can provide more information about the study, and answer any questions you may have.

*Please note that because we would like to interview a range of people depending upon certain characteristics (age, gender, types of health condition etc.) it may take a number of weeks before we can to arrange an interview date. In the event that we do not require your assistance, we will contact you to let you know.*

The interviews will take place either face-to-face or remotely using videoconferencing or telephone at a time convenient to you. The interview should last for around 60 minutes, although you can ask for a break or stop the interview at any time.

The interviewer will ask questions about your thoughts on how AI informed decision making (the use of computer programmes) compares with doctors making decisions about your health.

We will also show you an example of a patient with several long-term conditions and how their health care has been managed using a computer programme, for example, how the computer programme has helped decide what medications they may need. We will ask you what kinds of things are important to know if a computer is informing these kinds of decisions about the medications you are prescribed your health in general.

If you agree to take part, we would like to record the interview. After the interview, the recording will be written down (in a transcript) but all names and place names will be removed and no comments will be linked to you or any other person. The recording and transcript will be kept completely confidential and only the University evaluation team will have access to them.

At the end of the interview, you will be asked if you would be willing to be contacted at a later date to consider involvement in the second stage of the study. This stage will aim to explore preferences in terms of how computer programmes for AI directed clinical decision making are presented.

**What are the possible benefits of taking part?**

Although there is no direct benefit to you if you take part in an interview, the interviews will give us important information that should help patients with cMM along with the doctors who manage their conditions.

**Will I receive any financial reimbursement for taking part?**

You will be offered a £15 voucher (Amazon or Love 2 Shop) as a thank you for taking part in the interview.

**What are the possible risks and disadvantages of taking part?**

It may be upsetting to talk about living with multiple long-term conditions and your health care experiences. If this happens, we can stop or pause the interview at any time. We have put together a list of organisations that you can get in contact with who may be able to help. Many people have said that they find it helpful to talk to researchers about their experiences of living with conditions.

Taking part in the interview will take some of your time. However, you can choose a time and date that is suitable for you.

In light of COVID-19, we will adhere to all restrictions and recommendations. Support for participants will be provided to set up an online conferencing link, or by telephone according to participant preference. Researchers will have evidence of vaccination and/or negative results from a lateral flow test prior to interview if face-to-face.

**What if there is a problem?**

If you have any concerns, please get in touch with us using the contact details at the end of this information sheet. We will do our best to answer your questions. If your concerns are not addressed and you wish to make a formal complaint, you can refer to Dr Birgit Whitman (Head of Research Governance and Integrity at University of Birmingham [b.whitman@bham.ac.uk](mailto:s.jennings@bham.ac.uk)).

**Will my taking part in the study be kept confidential?**

All information collected about you during the research will be kept strictly confidential. Any paper information, like interview transcripts, will be stored in a secure and locked office at the University of Birmingham, and only accessed by designated members of the team (details

below). Any electronic information, like interview recordings, will be kept on secure servers at the University of Birmingham and protected by a password. All information will be anonymised so you will not be identified in any reports or publications that come from this research.

All audio-recordings will be kept for 10 years after the end of the study and then destroyed. Documentation and data from this part of the study will be securely stored at the University of Birmingham for 10 years.

**What will happen if I don’t want to carry on with the study?**

Your participation is completely voluntary. If you choose to take part but change your mind later, you are free to leave the study at any time, without giving a reason, and without your healthcare being affected. If you wish to withdraw from the study, please contact a member of the team (details are at the end of this document), However, please note that if you decide to withdraw more than 2 weeks after participating in an interview, any data already collected may still be used in the study.

**What will happen to the results of the research study?**

The information we collect will be analysed and the results will be presented in several ways:

- A short written summary of the results will be available on the OPTIMAL website hosted by the University of Birmingham, or can be sent out to participants should they prefer.
- A detailed report will be written and will be available for participants on the OPTIMAL website hosted by the University of Birmingham, or can be sent out to participants should they prefer.
- We will publish the results in academic journals.

Your details will not be shared at any time and you will not be identified in any of the results from the research.

**Who is organising and funding the research?**

The research study is funded by the National Institute for Health Research (NIHR). The research is managed at the University of Birmingham…….

**Who has reviewed the study?**

All research in the NHS is looked at by an independent group of people called a Research Ethics Committee (REC), to protect your interests. This study has been reviewed and given favourable opinion by __ Research Ethics Committee. Patients and public Involvement (PPI) members have been involved throughout all stages of the research study.

**What happens next?**

If you would like to participate in an interview, please contact a member of the research team whose details are given below.

**The research team:**

**For more information, contact:**
