# Supplementary material for: Exploring Patient Perspectives on the Use of Artificial Intelligence to Inform Joint Decision-Making for Patients With Multiple Conditions in Primary Care in the United Kingdom: Qualitative Study
Source: J Med Internet Res. 2026 Apr 21;28:e87507. doi: 10.2196/87507 (PMC13099014; doi:10.2196/87507)
Supplement: Multimedia Appendix 3 [file jmir-v28-e87507-s003.pdf]

OPTIMAL

IMPROVING THE LIVES OF PEOPLE LIVING WITH MULTIPLE LONG-TERM CONDITIONS

Could you help with our research?

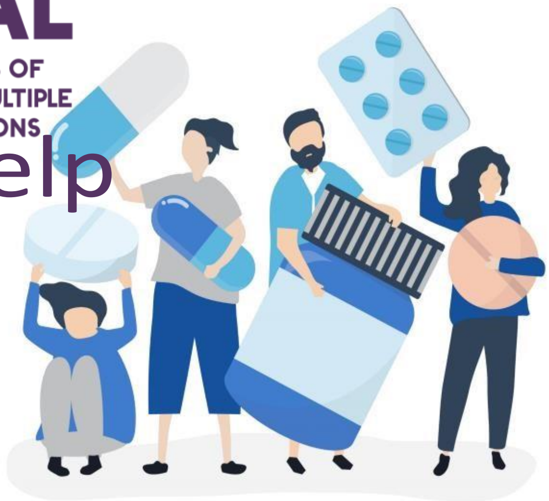

We want to speak to people who live with **two or more health conditions** about your healthcare experiences and how new technologies could affect treatments in future. If this is you and you'd be happy to take part in an interview, get in touch with Sarah at s.m.flanagan@bham.ac.uk or ***07966 679184***

What is this research?

We are carrying out interviews with people, lasting about an hour, to find out how they manage their health and how they would feel about the use of AI (artificial intelligence) in medical appointments in the future. Our research project (OPTIMAL) aims to understand how doctors can improve the information they give to patients when prescribing medicines, like how it might affect their future health.

Who can take part?

Anyone who has 2 or more long-term conditions. These are things like, high blood pressure, diabetes or depression. There are lots of different long-term health conditions and it doesn't matter which you have.
We want to hear a wide range of experiences, and we particularly encourage black and ethnic minority participants.

Participants will be offered a £15 Amazon or Love2Shop voucher as a thank you for taking part.

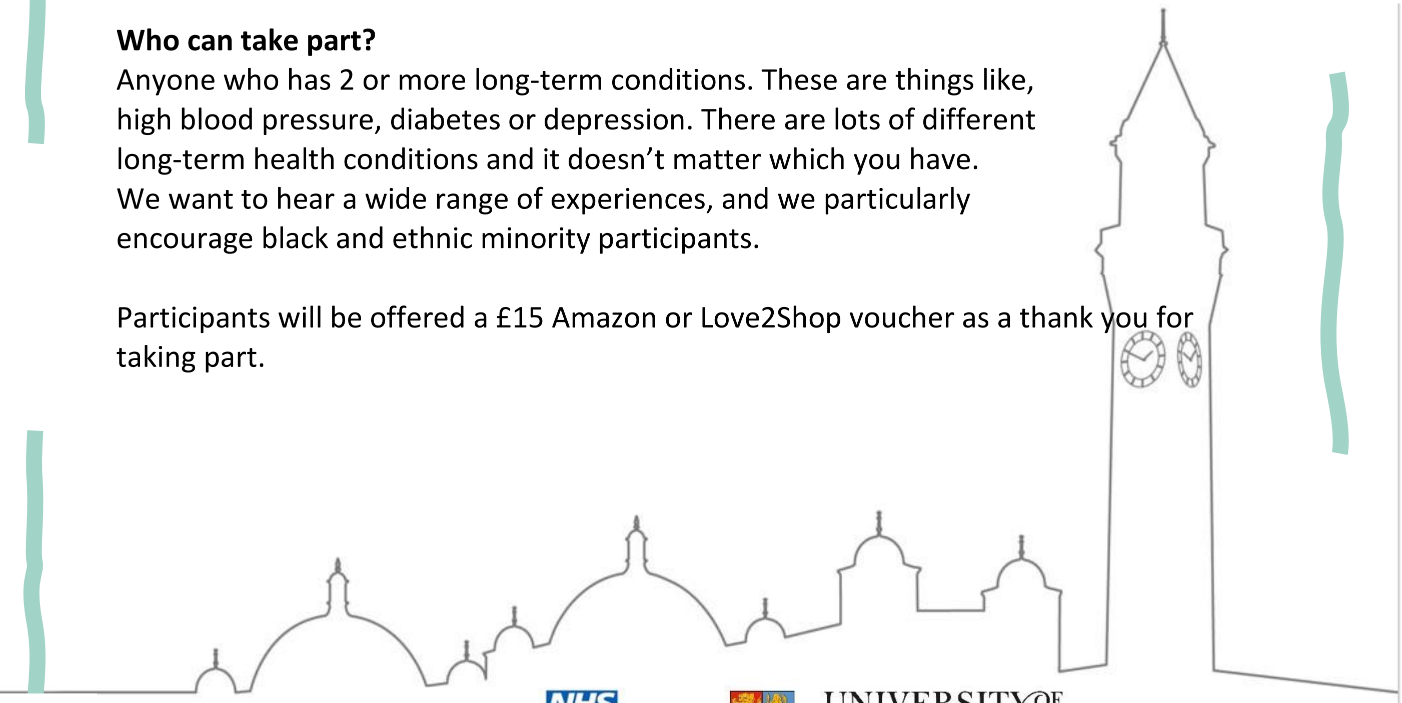

NHS National Institute for Health Research

UNIVERSITY OF BIRMINGHAM
