# Supplementary material for: Exploring Patient Perspectives on the Use of Artificial Intelligence to Inform Joint Decision-Making for Patients With Multiple Conditions in Primary Care in the United Kingdom: Qualitative Study
Source: J Med Internet Res. 2026 Apr 21;28:e87507. doi: 10.2196/87507 (PMC13099014; doi:10.2196/87507)
Supplement: Multimedia Appendix 4 [file jmir-v28-e87507-s004.docx]

Reflexivity statement:

Sarah Flanagan: Interviews were conducted by Sarah Flanagan, who was based at the University of Birmingham and working as a Research Fellow at the time of the study. She is a UK-based woman researcher with a PhD and has experience in qualitative research, including conducting interviews across multiple studies. Participants were not known to the interviewer prior to study commencement. Participants were informed about the aims of the research and were provided with an information document explaining AI and its potential use in healthcare. This document was reviewed by a patient advisory group to ensure clarity and accessibility.
